# The Clinical Significance of Endocannabinoids in Endometriosis Pain Management

**DOI:** 10.1089/can.2016.0035

**Published:** 2017-04-01

**Authors:** Jerome Bouaziz, Alexandra Bar On, Daniel S. Seidman, David Soriano

**Affiliations:** ^1^ Department of Obstetrics and Gynecology, The Chaim Sheba Medical Center, Ramat-Gan, Israel.; ^2^ Department of Urology, The Chaim Sheba Medical Center, Ramat-Gan, Israel.

**Keywords:** cannabis, endocannabinoid, endometriosis, pelvic pain

## Abstract

**Introduction:** Patients with endometriosis often suffer from diffuse and poorly localized severe pain. The current pain management strategies include medical and hormonal therapy, as well as surgery. Medical management of pain is often insufficient and is associated with high rate of recurrence. Better pain management is therefore of urgent need.

**Methods:** Among the various candidates, the endocannabinoid system (ECS) has recently emerged as a relevant pharmacological target for the management of endometriosis-related pain. A computerized literature search was performed to identify relevant studies combining the keywords “endometriosis,” “endocannabinoid,” “cannabinoid receptor,” “THC,” and “pain mechanisms.”

**Conclusions:** This review describes the multiple and complex pain mechanisms associated with endometriosis. Current data and theories concerning the link between the ECS and pain management for endometriosis patients are presented. Finally, we will discuss which aspects of endometriosis-associated pain can be targeted by modulation of the ECS.

## Introduction

Endometriosis is defined as the presence of endometrial glands or stroma in sites other than the uterine cavity, such as the ovaries, pelvic peritoneum, and the rectovaginal septum.^1^ This condition affects 5–15% of the women of reproductive age.^2^ Pain is one of the predominant clinical features of endometriosis. The patients often suffer from diffuse and poorly localized severe pain. It has an impact on the quality of life in many ways. Pain in endometriosis is often associated with psychological distress and fatigue, both of which may amplify pain.^3^ In more than 95% of cases, patients who suffer from deep infiltrated endometriosis (DIE) are prone to very severe pain. It can also include symptoms such as dysmenorrhea, dyspareunia, nonmenstrual pelvic pain, and, less commonly, dyschezia and dysuria.^4^

The management of hyperalgesia in endometriosis patients is a medical challenge.^1,3,5,6^ The current pain management strategies for endometriosis focuses mainly on medical treatments such as hormonal therapy, painkillers or nonsteroidal anti-inflammatory drugs (NSAIDs), and/or surgical resection. Medical management, however, is insufficient as it is associated with high rate of recurrence^7^ and imparts only partial relief of symptoms and/or chronic pelvic pain (CPP).^3,5,7^ Medical and surgical management also depend on whether the woman presently wants to conceive or not and if she may require an *in vitro* fertilization (IVF) treatment.

The pathogenesis of endometriosis remains unclear, although it is known that several pathways are involved. The endocannabinoid system (ECS) has emerged recently as an important factor in endometriosis development, maintenance, and pain mechanisms.^8–13^ These new revelations suggest that the ECS may potentially serve as a pharmacological target for endometriosis treatments, including pain management, and have a role in immune intervention and antiproliferative and antifibrotic effects.

In this review of the literature, we will first discuss the multiple and complex pain mechanisms involved in endometriosis and then analyze the existing data and theories concerning the link between pain management and ECS in endometriosis patients. Finally, we will review which aspects of endometriosis-associated pain can be targeted by modulation of the ECS.

## Methods

A computerized literature search was performed to identify relevant studies. We searched MEDLINE electronic databases (www.ncbi.nlm.nih.gov/sites/entrez) published between January 1994 and June 2015, combining the keywords “endometriosis,” “endocannabinoid,” “cannabinoid receptor,” “THC,” and “pain mechanisms.” Various combinations of the terms were used, depending on the database searched. We also examined reference lists for any studies not included in the initial literature search. However, it should be stated that this is not a systematic review.

## Pain Mechanisms in Endometriosis

The endometriosis-associated pain mechanisms are complex and interconnected and can be divided into three main categories of pain: the nociceptive, the inflammatory, and the neuropathic pains.

### Nociceptive pain

This type of pain occurs due to the activation of nociceptors, which are sensory receptors of the peripheral nervous system capable of transducing noxious stimuli (such as mechanical, chemical, and thermal) into energy, usually at a high threshold value. Depending on the location of the activated receptor, nociceptive pain may be visceral or superficial. The endometriotic lesions can cause mechanical nerve compression/infiltration and, thereby, activate the nociceptors.^14^ The visceral nociceptive fibers are thought to mediate the intensity of noxious stimuli when activated from cells in certain organs.^15^ Moreover, certain changes take place in the peritoneal fluid (PF) of the endometriosis patients. These changes involve an increase in the levels of cytokines, growth factors, and chemokines that can activate peripheral nociceptors^16^ and can sensitize the peripheral nerves through specific cell-surface receptors. As a result, the microenvironmental inflammatory response increases and pain is generated.^17,18^ The vanilloid receptor 1 (TRPV1) and other pain-producing agents have been found in the PF of endometriosis patients. These agents can directly cause an excitatory negative current value or affect the movement of ions and, thereby, influence endometriosis-associated pain.^19^

### Inflammatory pain

Inflammatory pain is a part of the nociceptive pain entity. The inflammatory mediators interact with the neurons to produce hypersensitivity and modify the perception of pain.^20^ Inflammation is involved in pain mechanisms as the nerve growth factor is upregulated by the inflammatory cytokines, tumor necrosis factor-α, and interleukin-1β. Endometriosis can be considered as a chronic inflammatory disease. Macrophages display features of alternative phagocytose of aged red cells and endometrial cell debris.^12^ This participates in the establishment of chronic local and general inflammatory environment. PF changes and specific hormonal phenotype during endometriosis create an environment of hypersensitivity to inflammatory stimuli. Moreover, significantly higher levels of advanced oxidation protein products were observed in perioperative PF samples of patients with DIE, which indicates the role of oxidative stress in the inflammatory process and development of endometriosis.^21^ In addition, the involvement of sphingosine pathway in the establishment of endometriosis can also not be ruled out.^22^

### Neuropathic pain

Neuropathic pain is associated with damage to the neurons themselves, following an infection or injury to the area, resulting in pain signals being sent to the central nervous system (CNS) regardless of noxious stimuli.^23^ Neuropathic pain is often described as “shooting pain,” as it travels along the nerves in an abnormal manner causing abnormal sensations of pain. The modification of the CNS has a key role in the experience of pain, especially CPP. Central sensitization occurs during endometriosis, and is associated with alterations in both the structure and function of the CNS.^15,23,24^ Regional modifications in the gray matter of the brain have been reported in patients with or without endometriosis and CPP, compared to healthy pain-free patients.^23^ Many of the symptoms associated with CPP that are attributed to endometriosis begin as early as adolescence or early adulthood.^25^ The central neurologic system is very ductile at young age,^26,27^ and hence, pain should be promptly treated to potentially decrease the impact of pelvic pain on brain development in adolescents.

## Multiple Psychological Effects on Pain Experience in Endometriosis

Nonexclusive to endometriosis, the general experience of pain—its severity and intensity and the effectiveness of treatments—depends on many psychological, cultural, and personal factors.^28^ In endometriosis, psychological factors may have an influence due to the nature of the disease and the impact it can have on women during childbearing years, especially dyspareunia and fertility concerns.^29^ Both issues can understandably cause anxiety and “pain catastrophizing” effect due to exaggerated negative response in the anticipation of pain that, in turn, amplify the pain experience.^30^ These symptoms can also impact self-esteem and relationships, causing additional worsening of the pain experienced by the patient.^31^

Now that we had a look at the pain mechanisms associated with endometriosis, we will focus on understanding the association of the ECS with the mechanisms of pain generated in endometriosis.

Going on, we must keep in mind that each patient's pain experience is different, because one pain mechanism may take dominance over the others. This could be because of differences in pathogenesis or disease entities, which mean symptoms, may only respond to certain treatments and at a certain point in the patient's menstrual cycle.^32^ These factors should be considered when developing strategies in personalized medicine to manage with more efficiency, the pain associated with endometriosis.

## Association Between the ECS and Endometriosis Pain

It has been recently shown that for endometriosis, the ECS, mainly known for their psychogenic effects, interacts with specific mechanisms associated with pain establishment, such as inflammation, cell proliferation and cell survival.^9–12,15,16,24,33^ These mechanisms play a key role in endometriosis-associated pain and in the establishment of the disease, its maintenance, and recurrence.

The ECS is defined as a group of endogenous cannabinoid receptors, ligands, and enzymes required for ligand biosynthesis and degradation, which are predominantly located in the brain, the CNS, and in the peripheral nervous system.^23,34^ As we have previously discussed, the mechanisms involved in endometriosis pain are both at central and peripheric neural levels. In addition, the ECS is involved in many physiological processes, including pain sensation, appetite, mood, and memory, and in mediating the psychoactive effects of cannabis.

Endocannabinoids are amides, esters, and ethers of long-chain polyunsaturated fatty acids, which act as lipid mediators. They bind to cannabinoid, vanilloid, and peroxisome proliferator-activated receptors. Endogenous ligands bind to the same receptors as the principal biologically active component of *Cannabis sativa*, Δ^9^-tetrahydrocannabinol (Δ^9^-THC).^35^ The two well-studied cannabinoid receptor ligands are anandamide (*N*-arachidonoylethanolamine; AEA) and 2-arachidonoylglycerol (2-AG). Two kinds of cannabinoid receptors are described: the cannabinoid 1 (CB1) and 2 (CB2) receptors. These receptors are membrane bound and usually found on the presynaptic neuron in the central and peripheral nervous systems.

CB1 receptors are highly expressed in the uterus, as well as in multiple nonreproductive tissues.^36^ The CB2 receptors are preferentially expressed abundantly in the immune system and intestines and in other tissues such as the lungs, uterus, pancreas, and skin.^9^ Studies have shown that human oocytes express CB1 and CB2 receptors and their localization varies during oocyte maturation stages.^37^ Moreover, the presence of AEA in the female reproductive tract fluids, as well as ovary, has been demonstrated.^38^ AEA plays an important role in folliculogenesis, preovulatory follicle maturation, oocyte maturation, and ovulation.^39^ A study conducted on women who had undergone IVF/intracytoplasmic sperm injection-embryo transfer showed that higher plasma AEA levels at ovulation and a significantly lower level during implantation are important for successful pregnancy.^40^ Furthermore, alterations in endocannabinoid signaling promote miscarriage in early pregnancy^41^ and this implicates the importance of ECS in female reproduction.

The levels of expression of CB1 and CB2 are variable during the menstrual cycle. Resuehr et al., described an important increase in CB1 receptors, messenger ribonucleic acid (mRNA), and protein in normal endometrial samples in the secretory phase, due to the ability of progesterone to regulate the receptor's expression.^36^ Progesterone exposure during the secretory phase (days 12–26 of cycle) is associated with upregulation of endocannabinoid receptors. It is suggested that this mechanism is triggered by injured endometrial tissue to control the nitric oxide (NO)-mediated inflammatory reaction, preventing degranulation and the release of pro-inflammatory mediators from human mast cells.^42^ In the research conducted by Resuehr et al.,^36^ the authors concluded that acute dioxin exposure resulted in failure of progesterone to upregulate endocannabinoid receptor CB1 expression in the endometrial cells.

Sanchez et al., conducted a study^10^ in which the authors compared the plasma levels of endocannabinoid ligands [AEA, 2-AG, *N*-oleoylethanolamine (OEA), and *N*-palmitoylethanolamine (PEA); OEA and PEA are the two AEA congeners and share biosynthetic and catabolic pathways with AEA]. They often accompany endocannabinoids in the tissues and the mRNA expression of some of the main receptors (CB1, CB2, TRPV1) in endometrial cells for women with or without endometriosis. They then analyzed the association between the levels and symptoms of endometriosis-associated pain. They discovered a significant increase in plasmatic endocannabinoid ligand with decreased local CB1 expression in women with endometriosis compared to those without endometriosis. This result suggests a negative feedback loop regulation, which may impair the capability of these mediators to control pain in endometriosis patients. Since the most common symptoms of endometriosis-associated pain are CPP, dysmenorrhea, and dyspareunia, the authors measured endocannabinoid ligands in women who experienced any of these symptoms. They also found elevated levels of AEA in women with moderate-to-severe dysmenorrhea (visual analog scale 51–100) and elevated PEA levels in women with moderate-to-severe dyspareunia (visual analog scale 51–100).

Some of the studies that we reviewed described endometriosis as an “endocannabinoid deficiency” condition, thereby partially explaining its implication with pain.^43^ Indeed, women with endometriosis have lower levels of CB1 receptors in endometrial tissue. Reduced ECS function has been suggested to lead to growth of endometriosis tissue and a more severe pain experience.^43–45^ Therefore, the ECS could be important for the establishment of pain associated with endometriosis.

## Effects of Synthetic Versus Natural Cannabinoid Agonists on Endometriosis

The main biologically active component of *C. sativa* is Δ^9^-THC. Many synthetic cannabinoid receptor agonists or antagonists have been developed, such as WIN 55212-2, AM-251, HU-210, JWH-133, and methanandamide. Although the clinical use of cannabinoid has been poorly studied, there is a growing body of evidence that suggests that both synthetic and natural cannabinoid receptor agonists have common interactions with inflammatory pain and neoangiogenesis. Indeed, cannabinoid receptor agonists and Δ^9^-THC modulate cytokine production, through upregulation of CB2 receptors, perhaps as a response mechanism triggered by injured endometrial tissue to control the NO-sustained inflammatory reaction. Cannabinoid receptor agonists and Δ^9^-THC modulate cytokines by binding to the CB2 receptors and prevent the degranulation and release of pro-inflammatory mediators from macrophages.^42^

WIN-55212-2, HU-210, JWH-133, and Δ^9^ THC, by binding to CB1 and/or CB2, inhibit vascular endothelial cell survival and migration as part of their antiangiogenic action.^46^ This is of importance in the growth of the endometriotic lesion and possibly in the mechanisms of neuropathic pain.

### Synthetic cannabinoid ligands

Most synthetic cannabinoid ligands act as agonists of the cannabinoid receptor CB1. Table 1 summarizes the mode of action of the cannabinoid ligands and their effect on endometriosis.

**Table 1.** **Biological Actions of Natural and Synthetic Cannabinoid Ligands on Endometriosis**

Cannabinoid ligands	Actions	Effect on endometriosis	References
WIN 55212-2	Agonist CB1/CB2	Decreased cell proliferation *in vivo* and *in vitro*	Leconte et al.^8^
AM251	Antagonist/inverse agonist CB1	Increased endometriosis-associated hyperalgesia	Dmitrieva et al.^11^
Methanandamide	Agonist CB1	Induced endometrial stromal cell migration	Gentilini et al.^12^
D9-THC	Agonist CB1/CB2	Induced migration of HEC-1B (human endometrial cell line)	McHugh et al.^55^

### Natural cannabinoid agonist

The main biologically active component of *C. sativa*, Δ^9^-THC, produces its effects through activation of G-protein-coupled cannabinoid receptors (CB1 and CB2). Few studies have evaluated the effect of cannabis consumption on endometriosis pain and evolution. One of the main limitations is the fact that it is, so far, impossible to know which molecules and doses have been used by the consumer. Therefore, the dose-related effect of cannabinoid consumption needs further investigation.

Although the consensus in current literature indicates that cannabinoids have antiproliferative effects on endometriosis, there are a few studies that have shown that Δ^9^-THC has a biphasic effect and that at lower concentrations (nanomolar range), it causes increased cell proliferation, while at higher concentrations (micromolar range) it decreases proliferation of cells. These studies were conducted on cancer cells.^47^ Moreover, it was demonstrated that Δ^9^-THC (50–100 nM) increases the proliferation and viability of androgen-independent PC-3 cells, which could also be true in the case of endometriosis.^9^

## Clinical Studies and Trials

Most of the clinical studies that focused on the relationship between ECS modulation and endometriosis-associated pain, analyzed the effect of PEA on pain.^33,48–53^ The PEA principle was first described in 1957 by Nobel laureate and Professor, Rita Levi-Montalcini.^54^

PEA is an endogenous fatty acid amide that binds to the peroxisome proliferator-activated receptor. PEAs affinity for CB1 and CB2 is not very strong. Hence, it has been suggested that PEA directly activates CB2-like receptor or potentiates endocannabinoid actions. The latter, also referred to as the “entourage effect,” is achieved through enhancing the endogenous anandamide activity by increasing the affinity for receptors and/or through reducing the enzymatic degradation of anandamide and is thereby considered a cannabinoid system modulator.

Table 2 shows the clinical studies published till date and their main results. Most of the studies used 400 mg of PEA with 40 mg of Polydatin. The results are encouraging regarding the improvement of pelvic pain, as all the clinical studies showed a statistically significant improvement in dysmenorrhea and CPP. The randomized control study conducted by Cobellis et al.,^49^ revealed better results than the placebo, but a less significant decrease in symptoms than with the use of NSAIDs. The use of NSAIDs, however, appears to present more side effects with long-term use and more contraindications.

**Table 2.** **Clinical Trials that Analyzed the Association Between Endocannabinoid System and Endometriosis-Associated Pain**

*n*	Design study and patient characteristics	Molecules tested and doses	Mode of delivery and length	Results of the studies	References
220	RCT	PEA 400 mg and Polydatin 40 mg versus placebo	Once a day. Ten days a month	Improvement of pelvic pain in 98.18%	Tartaglia et al.^56^
	Primary dysmenorrhea			The combination of PEA and transpolydatin was more effective than placebo (*p*<0.001)	
	Age 16–24			No side effect	
	Not necessarily endometriosis but all etiology of dysmenorrhea				
56	Endometriosis	PEA 300 mg and LA 300 mg	Twice daily for 9 months	Progressive reduction of the pain syndrome	Caruso et al.^48^
	CPP			Improve the QoL	
	Quality of life			Improve sexual life of women	
	Sexual health				
61	Endometriosis	Group A (*n*=21): PEA 400 mg + transpolydatin 40 mg	Group A: twice a day for 3 months	All groups: decrease in dysmenorrhea, dyspareunia, and pelvic pain	Cobellis et al. ^49^
	RCT	Group B (*n*=20): placebo	Group B: placebo 3 months	PEA + transpolydatin more effective than placebo (*p*<0.001)	
	Three groups after laparoscopy conservative treatment of endometriosis	Group C (*n*=20): celecoxid 200 mg	Group C: twice a day for 7 consecutive days	Celecoxid best decrease compared to placebo and PEA	
24	Endometriosis	PEA 400 mg	Twice a day for 90 days	Statistically significant improvement of pelvic pain, dysmenorrhea, and dyspareunia	Lo Monte et al.^50^
	CPP	Polydatin 40 mg		Improvement in QOL	
	Evaluation of the use of associated pain killer			Not statistically significant for dysuria and dyschezia	
				Decrease assumption of NSAIDs	
47	Endometriosis Group A: rectovaginal nodule Group B: ovary	PEA 400 mg Polydatin 40 mg	Twice a day for 90 days	Intensity of endometriotic pain (dysmenorrhea, chronic pelvic pain, dyspareunia, and dyschezia) decreased significantly for both groups (*p*<0.0001)	Giugliano et al.^33^
				Pain intensity decreased equally in the two groups except for dysmenorrhea, which was reduced more rapidly in group B.	
				Efficacy of drugs after 30 days	

*n*, No. of patients included in the study; CPP, chronic pelvic pain; NSAID, nonsteroidal anti-inflammatory drug; PEA, *N*-palmitoylethanolamine; QOL, quality of life; RCT, randomized control study.

**FIG. 1.**
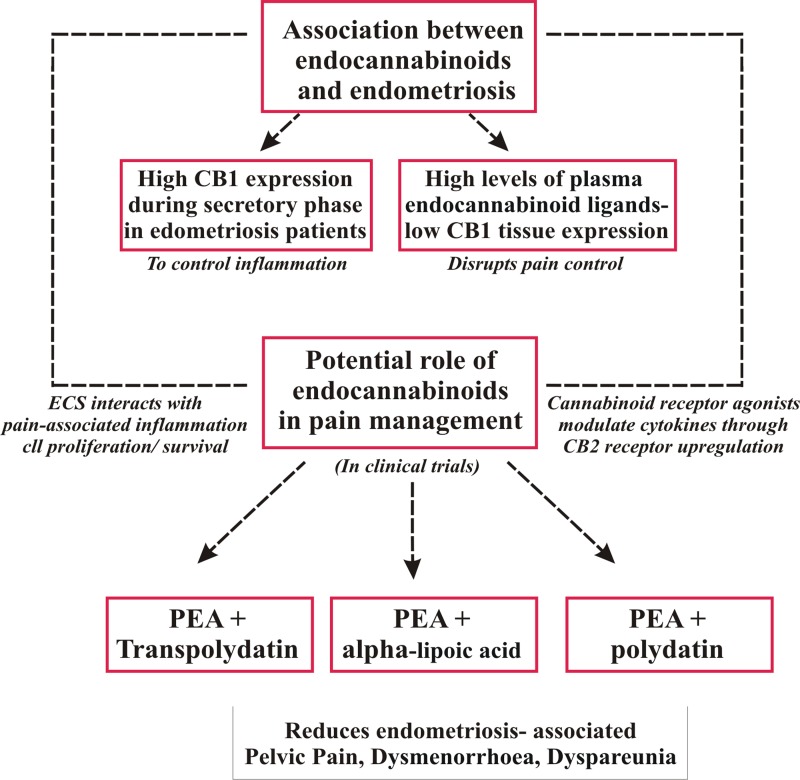
The different implications of ECS in endometriosis and pain. High levels of CB1 receptors and endocannabinoid ligands have been observed in endometriosis patients. Although this occurs as a mechanism to reduce inflammation, low CB1 levels in the tissue act as a negative feedback loop and disrupt the pain mechanism. However, several natural and synthetic agonists modulate ECS and reduce endometriosis-associated pain. ECS, endocannabinoid system; PEA, *N*-palmitoylethanolamine.

**FIG. 2.**
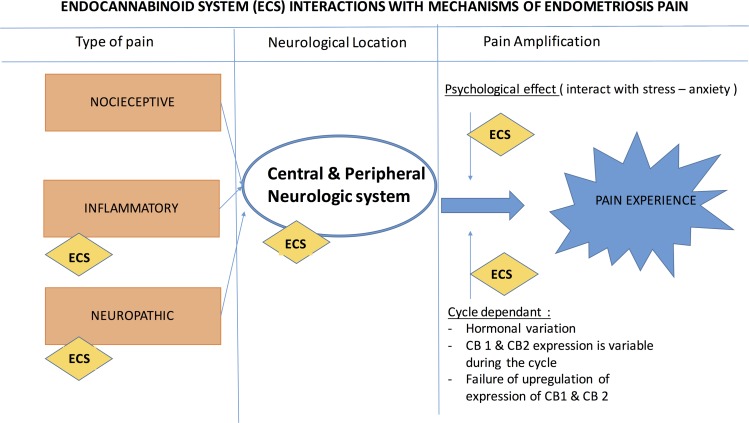
ECS interactions with mechanisms of endometriosis pain.

Results regarding dyspareunia and sexual life are encouraging. The studies that analyzed “sexual life of women” or “dyspareunia”^33,49,50^ showed a significant improvement with the use of PEA. One study^33^ evaluated the delay of action on the symptoms studied, and the therapy appeared to continue to be effective up to 30 days after treatment. Lo Monte et al.^50^ did not find a statistically significant improvement in dysuria and dyschezia. One hypothesis that explains this result is that the lesions causing dysuria and dyschezia may be too advanced and unreceptive to medical treatment. Another hypothesis is concerned with the role of the peripheral nervous system in modulating endometriosis-related pain by the so-called “pelvic-lower abdominal cross-organ sensitization” among the gastrointestinal, urinary, and gynecological visceral locations. The dichotomizing fibers process, in which the endings of one neuron supply nerves to two different tissues, may explain the second hypothesis. This process suggests an anatomical and physiological basis for referred pain and may partially explain why CPP and its common comorbidities such as bladder pain or irritable bowel syndrome occur in women with endometriosis. Another notable point is that none of these studies reported adverse side effects. A limitation that has been proposed for the use of cannabinoids in pain management is the fear of drug dependence due to smoking and toxicity. In such cases, alternate drug delivery routes that have low side-effects could be considered. A schematic diagram which explains the multiple actions of ECS on endometriosis and pain has been provided (Figs. 1 and 2).

## Need for Investigation and Caution in Endometriosis Pain Management

### Better characterization of pain

As we have seen in this review, the mechanisms of pain associated with endometriosis are many and complex. To provide more effective therapies, we must be able to better define the kind of pain involved when we treat patients. To undertake research, it is mandatory to have a common terminology to be able to compare what is comparable.

### Phenotype of the patient

A patient's phenotype is still commonly used in clinical practice. Hormonal concentrations, cannabinoid receptor levels, and cannabinoid agonists' levels in the blood and the PF must be better evaluated. Indeed, the knowledge of the intervariability of these components, along with consideration of the variability of pain during the menstrual cycle, in patients can lead to a more personalized management of pain.

### Investigate the effects on the habitual consumers of cannabis

As in cancer biology research, we must understand which kind of cannabis has stronger pain alleviating benefits and fewer side effects. Many types of cannabis exist, each with different active molecules, varying levels of blood concentrations, and psychoactive effects. We have to understand if consumers are using cannabis more for its central or peripheral effects.

### Impact of psychological effects

Some psychological effects of THC (difficulty in concentration, sleepiness, etc.) are not compatible with occupational activities. Thus, research on targeting treatments should focus on avoiding these effects.

## Conclusions

The interactions between the ECS and pain associated mechanisms in endometriosis patients occur at several levels: changes in central and peripheral neural system, involvement of neuropathic and inflammatory pain, psychological interaction with the pain experience, hormonal variability of the pain, and the expression of cannabinoid receptors, enzymes, and ligands.

Pain management for patients with endometriosis needs to be more effective, target the hormonal and immunologic environment, downregulate proliferation while enhancing apoptosis, and normalize the invasive mechanisms and neuroangiogenesis processes. ECS modulation appears to be a good therapeutic strategy by potentially combining all these factors.

Targeting endocannabinoid modulation to treat pain is probably more than just treating the pain as it may impact several levels of the pathogenesis and the proliferation of the disease. Special attention and further investigation are needed to evaluate the impact of the potential therapeutic side effects, especially on fertility and pregnancy outcomes.

## Abbreviations Used

2-AG: 2-arachidonoylglycerol
AEA: *N*-arachidonoylethanolamine
CB: cannabinoid
CNS: central nervous system
CPP: chronic pelvic pain
DIE: deep infiltrated endometriosis
ECS: endocannabinoid system
NO: nitric oxide
NSAID: nonsteroidal anti-inflammatory drug
PF: peritoneal fluid
THC: tetrahydrocannabinol
